# Case report: Miliary tuberculosis complicated by pediatric acute respiratory distress syndrome in a 12-year-old girl

**DOI:** 10.3389/fped.2023.1189838

**Published:** 2023-09-01

**Authors:** Jiarui Zhu, Ning Chen, Yunxiao Shang, Yong Feng

**Affiliations:** Department of Pediatrics, Shengjing Hospital of China Medical University, Shenyang, China

**Keywords:** miliary tuberculosis, pediatric acute respiratory distress syndrome, interferon-γ release assay, child, diagnosis

## Abstract

Acute respiratory distress syndrome (ARDS) is a rare complication of miliary tuberculosis, particularly in pediatric patients. Comorbidities and delayed diagnosis can worsen the prognosis of patients with miliary tuberculosis. A 12-year-old girl presented with fever for 20 days, and cough and tachypnea for 4 days. She was diagnosed with miliary tuberculosis complicated by pediatric ARDS. She had atypical clinical manifestations and imaging findings, a negative contact history, and negative results of a tuberculin skin test (TST) and T-SPOT.*TB*. Diagnostic bronchoscopy and bronchoalveolar lavage helped make the diagnosis of tuberculosis. Effective treatment was promptly initiated after confirmation of the diagnosis, and the patient's condition improved. This case illustrates that a negative contact history and laboratory results cannot rule out tuberculosis. False-negative TST and T-SPOT.*TB* results should be evaluated carefully. Bronchoscopy may be useful for identifying pathogens in patients with pneumonia of unknown etiology, and corticosteroids should be administered with caution.

## 1. Introduction

Tuberculosis (TB) remains a major public health concern worldwide although it is preventable and curable. According to data from the World Health Organization, of the estimated 1.1 million children (aged < 15 years) who develop TB annually, an estimated 226 000 (approximately 20.5%) die of TB, suggesting that TB has a high mortality rate in children (1). Miliary TB is caused by the hematogenous spread of *Mycobacterium tuberculosis* (Mtb) and is more common in children than that in adults, especially infants (2), and has a high case fatality rate.

Acute respiratory distress syndrome (ARDS) owing to diffuse alveolar damage is a rare complication of miliary TB (3). To our knowledge, only five children (<15 years) with miliary TB and pediatric ARDS (PARDS) have been reported since 1995 (4–7). This severe comorbidity causes critically severe illness with a high mortality rate. The diagnosis of miliary TB can be delayed owing to the various clinical manifestations of PARDS, leading to a poor prognosis. Piastra et al. (5) reported 3 cases of miliary TB complicated by PARDS. The patients were critically ill and had severe complications such as pneumothorax and septic shock. Herein, we report the case of a 12-year-old girl diagnosed with miliary TB complicated by PARDS. In contrast to previous cases, our patient had occult onset without clear evidence from her contact history, imaging, TB diagnostic tests, or clinical manifestations, leading to difficulties in making the diagnosis.

## 2. Case presentation

**Figure 1:**
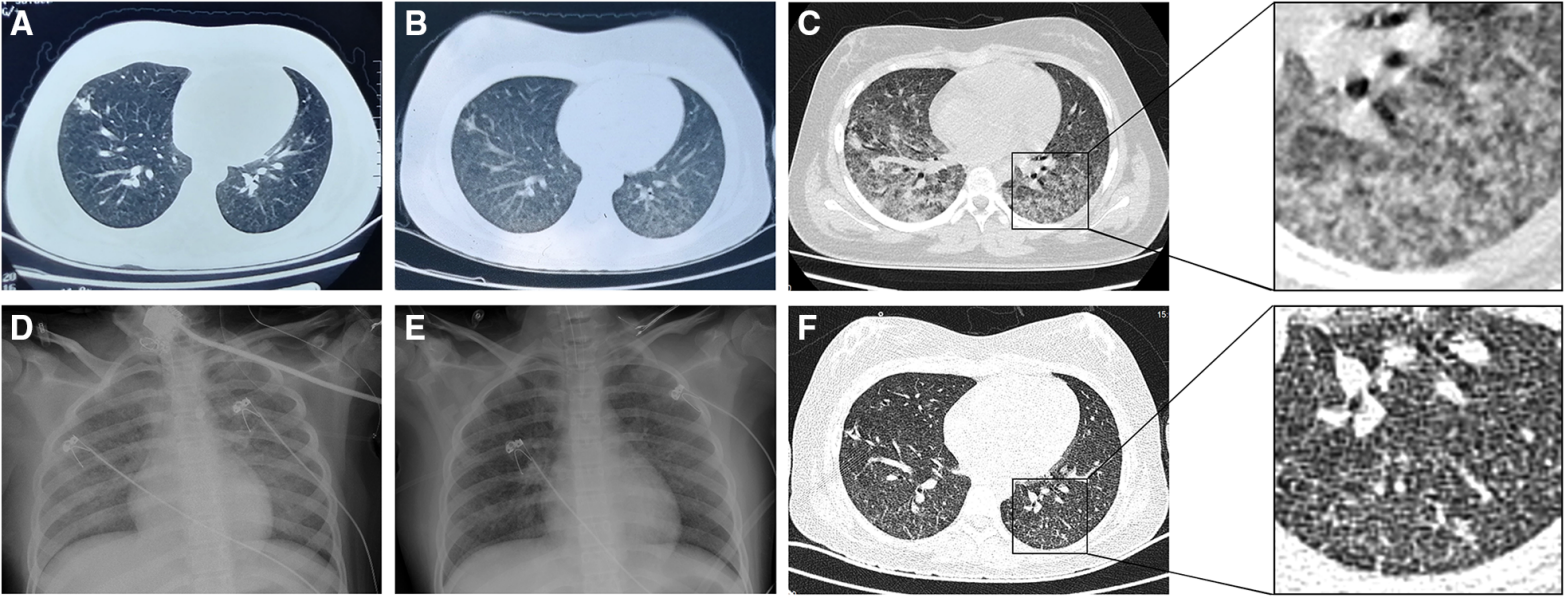
Chest imaging of the patient. (**A**) Computed tomography **(**CT) 14 days before admission showing an ill-defined nodular opacity in the lower lobe of the right lung. (**B**) CT 7 days before admission showing increased infiltrates in both lungs. (**C**) CT on day 3 after admission showing ground-glass opacities in both lungs with increased infiltrates and partial consolidation. (**D**) Radiography on day 5 after admission showing bilateral diffuse ground-glass changes (opacification and increased lung markings), in keeping with acute respiratory distress syndrome. (**E**) Radiography on day 8 after admission showing improvement of the ground-glass changes. (**F**) CT on day 12 after admission showing improvement of the ground-glass opacities and a few scattered nodules.

A previously healthy 12-year-old girl presented to our hospital with a fever for 20 days and cough and tachypnea for 4 days. The patient had first visited a local hospital with intermittent fever (maximum: 39.4°C, once or twice daily). Routine blood test results and the C-reactive protein level were normal. The titer of *Mycoplasma pneumoniae* (MP) immunoglobulin M (IgM) was 1:80. Chest computed tomography (CT) revealed an ill-defined nodular opacity in the lower lobe of the right lung (Figure 1A). The patient was treated with azithromycin and erythromycin for 7 days without any improvement. Considering the possibility of refractory MP pneumonia, dexamethasone was administered for 3 days, and the fever resolved. However, the patient became febrile again following dexamethasone discontinuation. Subsequently, she was admitted to another hospital with recurrent fever. Repeat chest CT showed increased infiltrates in both lungs (Figure 1B). She was treated with cefotaxime for 6 days and azithromycin for 3 days, but her fever did not improve, and she developed a paroxysmal dry cough and dyspnea. An interferon-γ release assay was performed using T-SPOT.*TB*, and the results were negative. (The mixed lymphocyte culture count was 3.2 × 10^6^/L). The patient was transferred to our hospital for further diagnostic workup and treatment.

On admission, her vaccinations were up to date. Her TB contact history was carefully evaluated, and no known or suspected exposure was identified. Physical examination revealed a body temperature of 36.5°C, blood pressure of 96/73 mmHg, pulse rate of 138 beats/min, respiratory rate of 58 breaths/min, and oxygen saturation of 85% breathing ambient air. Her growth parameters were normal for her age. Chest auscultation revealed moist rales in both lungs, with normal vocal and tactile fremitus. She had no palpable superficial lymph nodes. Abdominal examination revealed no abdominal tenderness, distention, masses, abdominal wall rigidity, or diminished bowel sounds. Central nervous system examination revealed normal deep tendon reflexes, a normal Babinski response, negative Kernig sign, negative Brudzinski sign, and no neck stiffness. The rest of the physical examination findings were unremarkable.

Blood tests performed on admission showed a white blood cell (WBC) count of 4.7 × 10^9^/L with 65.7% neutrophils and 23.7% lymphocytes, a normal C-reactive protein level, and a mildly elevated procalcitonin level (0.12 ng/ml; normal: <0.05 ng/ml). Her liver and kidney function, lactate dehydrogenase level, and urine routine test results were normal. Arterial blood gas analysis revealed a pH of 7.41, PaCO_2_ of 35 mmHg, and PaO_2_ of 118 mmHg on fraction of inspiration oxygen (FiO_2_) of 40% breathing via a face mask. Serum antibody tests revealed an MP IgM level of 1.06 S/CO (normal: <0.8 S/CO) and *Chlamydia pneumoniae* IgM of 1.49 S/CO (normal: <0.9 S/CO). However, neither MP nor *Chlamydia pneumoniae* were considered as the cause of the pneumonia because the patient had received macrolides for a week without any improvement. Serum antibody tests for cytomegalovirus IgM, Epstein–Barr virus IgM, and early antigen (EA)-IgG and herpes simplex virus IgM were negative. Polymerase chain reaction (PCR) tests of nasopharyngeal swabs for adenovirus, respiratory syncytial virus, and influenza virus were negative. Human immunodeficiency virus (HIV) serology and bacterial culture of blood were negative. Based on the clinical manifestations, physical examination findings, and the laboratory test results, the patient was initially diagnosed with acute severe pneumonia and hypoxemia. Oxygen supplementation (5 L/min via a mask) was administered to maintain normal oxygen saturation. She was treated with azithromycin and ceftazidime as antibiotic therapy and intravenous immunoglobulin as supportive therapy.

On day 3, the patient's WBC count decreased to 2.7 × 10^9^/L with an absolute lymphocyte count of 0.4 × 10^9^/L. Because of the unknown etiology and leukopenia, lymphocyte subsets were analyzed. This revealed a total T cell count of 209/μl (normal range: 690–2540/μl), CD8^+^ T cell count of 85/μl (normal range: 190–1140/μl), CD4^+^ T cell count of 92/μl (normal range 410–1590/μl), NK cell count of 25/μl (normal range: 90–590/μl), and total B cell count of 179/μl (normal range: 90–660/μl). The tuberculin skin test (TST) was negative. In the afternoon, the patient's dyspnea worsened. Her oxygen saturation was 83% on 60% FiO_2_ via a facemask, and arterial blood gas analysis revealed a pH of 7.45, PaCO_2_ of 47 mmHg, PaO_2_ of 81 mmHg, and PaO_2_/FiO_2_ ratio of 135 mmHg. She was transferred to the pediatric intensive care unit. Chest CT revealed ground-glass opacities in both lungs with increased infiltrates and partial consolidation (Figure 1C). She was diagnosed with PARDS and underwent noninvasive ventilation [BIPAP mode, peak inspiratory pressure/positive end-expiratory pressure (PIP/PEEP) = 29/8 cmH_2_O, FiO_2_ = 70%]. *Legionella* IgG test results were negative. Her sputum tested negative for SARS-CoV-2 using reverse-transcription PCR (RT-PCR). The antimicrobial therapy was changed to levofloxacin, cotrimoxazole, ribavirin, and caspofungin to cover uncommon pathogens.

**Figure 2:**
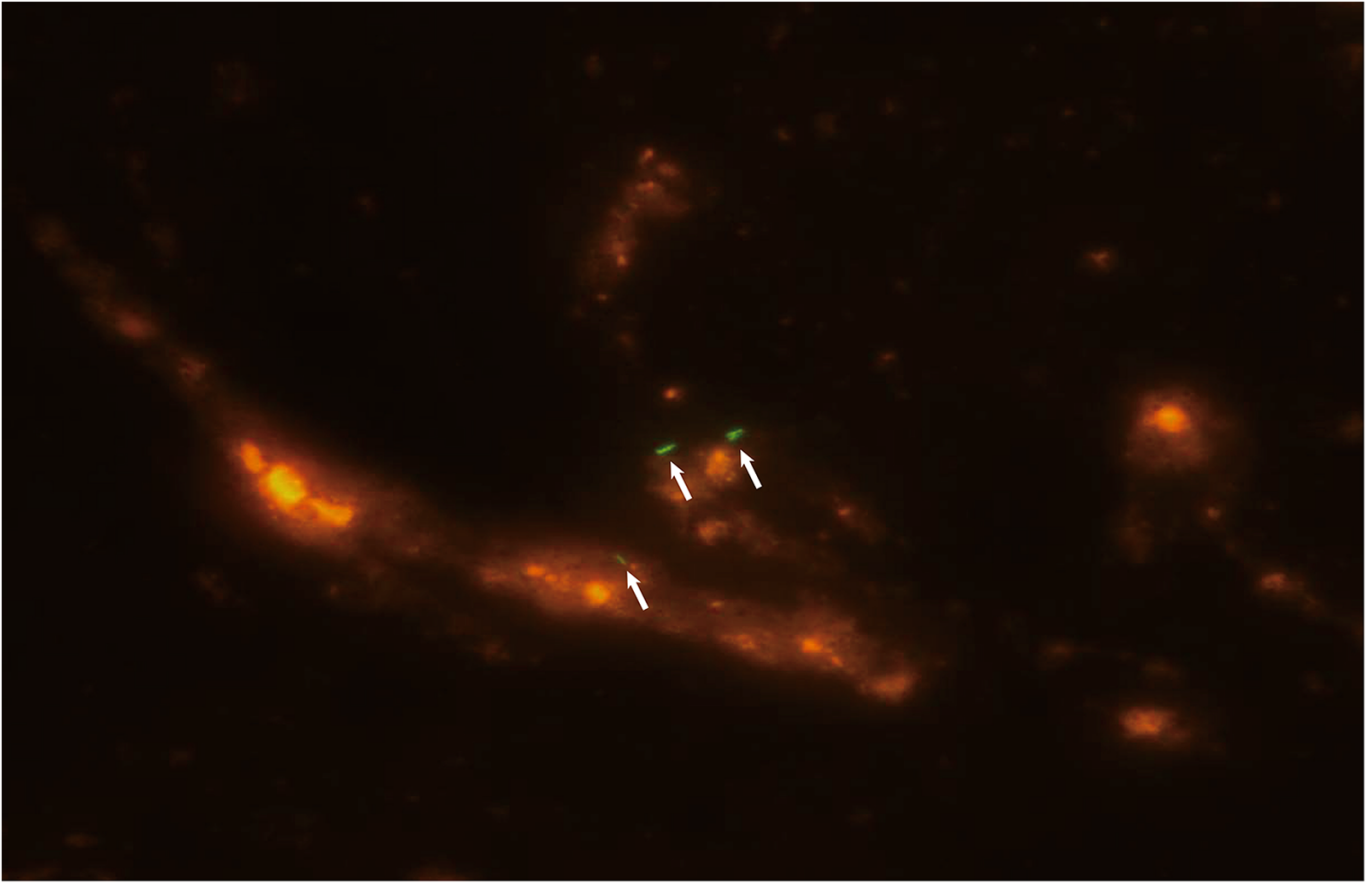
Fluorescent staining of a smear of bronchoalveolar lavage fluid showing acid-fast bacilli (white arrow) consistent with *Mycobacterium tuberculosis*.

Diagnostic bronchoscopy was performed on day 5 using flexible bronchoscopy (Olympus, Tokyo, Japan) under deep sedation with midazolam premedication and local anesthesia. Pediatric bronchoscopes were used (external diameter: 2.8 mm). The procedure was performed nasally and included an upper and lower airway evaluation. Macroscopic findings included thick mucus secretions, mucosal hyperemia, and edema. Routine bronchoscopic alveolar lavage was performed and bronchoalveolar lavage fluid (BALF) was collected from the lesion. During the procedure, the patient developed dyspnea and decreased oxygen saturation. The procedure was suspended and supplemental oxygen was administered. The procedure was continued after the patient's condition recovered. Her dyspnea worsened after the bronchoscopy. She was intubated and underwent mechanical ventilation (BIPAP mode, PIP/PEEP = 28/10 cmH_2_O, FiO_2_ = 70%). Chest x-ray showed bilateral diffuse ground-glass opacities consistent with ARDS (Figure 1D). Mtb smear (Figure 2) and DNA analysis of the BALF were positive. Tests for rifampicin resistance showed negative result. Metagenomic next-generation sequencing (mNGS, Genskey Medical Technology Co., Ltd, Beijing, China) of the BALF identified 16 of 420842 RNA sequence reads corresponding to the Mtb complex. There was no sequence read corresponding to other potential pathogens. (The methods and quality control of mNGS are described in Supplementary File S1). The T-SPOT.*TB* assay was repeated and found to be positive, and the mixed lymphocyte culture count was 51 × 10^6^/ml. The absolute lymphocyte count was 0.7 × 10^9^/L. The patient was diagnosed with TB. The antimicrobial therapy was changed to an antitubercular treatment (ATT). As ethambutol has not been approved by the China National Medical Products Administration for treating children aged under 13 years and the patient's parents declined to provide informed consent because of concerns about the toxicity, ATT was initiated with a three-drug regimen (isoniazid, rifampicin, and pyrazinamide) instead of the standard four-drug regimen (isoniazid, rifampicin, pyrazinamide, and ethambutol) (8–10). After starting ATT, the patient's severity and frequency of fever gradually decreased; her vital signs improved, and the FiO_2_ was gradually reduced.

Repeat chest radiography on day 8 showed improvement in the ground-glass changes (Figure 1E). Because of the possibility of hematogenous spread of Mtb, renal and cardiac ultrasound were performed, and the results were normal. Lumbar puncture revealed elevated intracranial pressure (280 mmHg) with normal cerebrospinal fluid cell counts, and mannitol was administered. On day 12, chest CT was repeated, revealing improvement in the ground-glass opacities and a few scattered nodules (Figure 1F). The patient was diagnosed with miliary TB. Two days later, the absolute lymphocyte count was 1.3 × 10^9^/L (46.2% T cells and 44.7% B cells), and the CD4^+^ T cell count was 219/μl, which was more than two-fold higher than that on day 3. The patient was previously healthy without HIV infection or a family history of immunodeficiency. Therefore, we considered that the decreased lymphocyte count was attributable to Mtb infection and the immunodeficiency was excluded. The patient's oxygen saturation level improved to 92% on ambient air. She was weaned off the supplementary oxygen and was discharged 17 days after admission. After the diagnosis of TB, her parents also underwent chest CT and sputum examination for Mtb, which were normal.

## 3. Discussion

Miliary TB complicated by ARDS is rarely reported in children. To our knowledge, only five cases in children (aged < 15 years) have been reported since 1995 (4–7). Of the five cases, four were in patients aged less than 5 years, of whom two were infants. ARDS is reported more frequently in adults, mostly in middle-aged and older adults (11–14). The low incidence of ARDS in older children, than in younger children and adults, may be attributable to an immature immune response in young children and an age-related decline in cellular immunity in adults (13, 15). However, for unknown reasons, another peak in incidence occurs during late adolescence and early adult life (13). Our patient was a 12-year-old adolescent, which is an age with a relatively high TB incidence. Based on her worsening dyspnea, increased infiltrates on chest CT, and PaO_2_/FiO_2_ of 135 mmHg, she was diagnosed with PARDS according to the Pediatric Acute Lung Injury Consensus Conference 2015 and 2023 guidelines (16, 17). Currently, it is believed that miliary TB can lead to PARDS in several ways, including the effect of lipoarabinomannan (12, 18) and inhaling large amounts of tuberculin and tuberculosis proteins within a short period (11, 14). However, the intrinsic mechanism remains unclear, and further studies are needed.

A history of TB contact is important for diagnosis; however, as illustrated by this case, a lack of history of TB contact does not rule out the possibility of TB. Consistent with our case, none of the five reported pediatric cases had a history of TB contact (4–7). A significant proportion of children with TB have no history of contact (19, 20). The probability of known contact increases with age (21, 22). Preschool children are generally exposed in the household, whereas older children are exposed to more people and can be exposed at home and in the community (21–23). China is a country with high TB burden, and children are at high risk of contracting TB. Our patient was a 12-year-old girl from China who might have had contact with TB in daily life.

T-SPOT.*TB* has a higher specificity than the TST, but either test can be used to diagnose TB infection (24). However, false-negative TST and T-SPOT.*TB* results can occur, particularly in immunocompromised individuals (25). Approximately 10% of cases of TB have false-negative TST and T-SPOT.*TB* results (25, 26). In our case, the TST and T-SPOT.*TB* results were initially false-negative, possibly owing to the patient's low CD4^+^ T cell count. Previous studies have shown that a decline in CD4^+^ T cell count increases the false-negative rates of TST and T-SPOT.*TB* (27, 28). The patient's relatively severe condition and use of corticosteroids suppressed her cellular immune response, leading to a low T lymphocyte count, including a low CD4^+^ T-cell count. Her second T-SPOT.*TB* result, performed after her lymphocyte count had increased, was positive, suggesting that false-negative T-SPOT.*TB* results may be related to decreased cellular immune function. Although we performed lymphocyte subset analysis, this is not required for all cases of unexplained pneumonia. The timely diagnosis in our case depended on BALF evaluations using multiple diagnostic tools, including culture, DNA testing, smear examination, and mNGS. mNGS has superior analytical performance in terms of sensitivity using X-pert MTB/RIF and culture as the comparators and a substantially short turnaround time for Mtb detection (29). Mtb sputum smears provide the simplest and most direct evidence of Mtb infection. In our case, it was difficult to obtain sputum because the patient had a nonproductive cough. Therefore, when the patient did not respond to several different empirical antibiotics, diagnostic bronchoscopy was performed to collect samples from the lower respiratory tract. However, bronchoscopy is not recommended for routine TB diagnosis in children as it is invasive, costly, and can cause various adverse effects (30).

Typical imaging findings of miliary TB include miliary nodules characterized by a uniform size, distribution, and density. The miliary nodules may be obscured by infiltrating ground-glass opacities of ARDS (31–33). In this case, the patient already had infiltrates on chest CT before admission, which may have been an early sign of PARDS. On day 3 of hospitalization, chest CT showed ground-glass opacities in both lungs with increased infiltrates, suggesting the occurrence of PARDS. After effective treatment, the ground-glass opacities improved, and a few scattered nodules were observed, which are not typical images of miliary tuberculosis. Miliary TB and ARDS are associated with diffuse alveolar damage, and miliary TB is strongly associated with ARDS (3). Diffuse alveolar damage is the most common histological feature of ARDS. Miliary TB can cause acute exudative hypersensitivity and capillary endothelial injury, leading to pulmonary edema and hyaline membranes, which are the pathological changes associated with ARDS (11, 12, 14, 18). Notably, imaging of miliary TB may be obscured by changes due to ARDS.

The early use of corticosteroids might have led to the dissemination of Mtb, as in this case. Before the onset of PARDS and the diagnosis of TB, our patient was treated with dexamethasone for 3 days. Subsequently, her CD4^+^ T cell count decreased to 92/μl, and chest CT showed increased infiltrates of both lungs. After effective treatment, her condition improved, and her CD4^+^ T cell count increased to 219/μl, although it remained below normal. This and several previously reported cases (32–35) suggest that dexamethasone may suppress cellular immune function, leading to a decline in resistance to Mtb and the dissemination of Mtb. CD4^+^ T cells play a crucial role in the containment of Mtb (36). Corticosteroids may have an adverse effect by decreasing the number of CD4^+^ T cells (37), leading to the proliferation and dissemination of Mtb and the development of miliary TB. Therefore, corticosteroids should be used cautiously in cases of fever of unknown etiology.

After the diagnosis of pulmonary miliary TB, the possibility of hematogenous spread of Mtb to the renal, cardiac, and central nervous systems was evaluated. We did not perform ultrasonography abdomen as the patient did not have symptoms or signs of abdominal TB. Ultrasonography abdomen is a simple and non-invasive examination that plays an important role in the diagnosis of abdominal TB (38–40). Therefore, when encountering patients with miliary TB, ultrasonography abdomen should be considered, regardless of whether the patient has abdominal signs. According to the World Health Organization (WHO) guidelines, children and adolescents with severe pulmonary disease should be treated with a four-drug regimen for 2 months followed by a two-drug regimen for 4 months at standard dosages (8). This patient had severe miliary TB; thus, according to the WHO guidelines, a four-drug regimen should have been used as the initial treatment. However, because the China National Medical Products Administration does not recommend ethambutol for children under the age of 13 years and the patient's parents declined consent for ethambutol treatment, our patient was treated with a three-drug regimen. The three-drug regimen should not be routinely used as initial ATT in severe cases.

In conclusion, the combination of miliary TB and PARDS is rare. It is life-threatening and easily misdiagnosed. TB contact evaluation, TST, and T-SPOT.*TB* are vital for the diagnosis; however, negative results do not rule out the diagnosis. Bronchoscopy may help identify pathogens when necessary. Corticosteroids should be used with caution in patients with fever of unknown origin.

## Data Availability

The original contributions presented in the study are included in the article/**Supplementary Material**, further inquiries can be directed to the corresponding author.
