# Supplementary material for: Case report: Miliary tuberculosis complicated by pediatric acute respiratory distress syndrome in a 12-year-old girl
Source: Front Pediatr. 2023 Sep 1;11:1189838. doi: 10.3389/fped.2023.1189838 (PMC10507688; doi:10.3389/fped.2023.1189838)
Supplement: Supplementary file 1 [file Datasheet1.pdf]

Supplementary Material

Procedure and quality control of mNGS

1 Procedure of mNGS

Bronchoalveolar lavage fluid (BALF) sample was collected from patients according to standard procedures. The sample was stored in a sterile container, then was preserved and transported in dry ice. The whole process of sequencing and pathogen detection pipeline were carried out in the laboratory of Tianjin Genskey Medical Technology Co., Ltd.

BALF was centrifuged at 8000 g for 5 min, and the pellet was resuspended in lysis buffer. Then, 1.5-mL microcentrifuge tubes with 0.5 mL of sample and glass beads were vortexed vigorously at 3000 rpm for 30 min.After bead-beating, 0.3 mL of the sample was separated into a new 1.5-mL microcentrifuge tube for DNA extraction. Concentration of DNA was measured by a Qubit Fluorometer. Total 100 ng of DNA was subjected to library construction by a transposase-based methodology. After purification and size-selection, the concentration of library was determined again by a Qubit instrument. Pooled samples were sequenced on a illumina Nextseq 550 (Illumina Inc., San Diego, CA, USA) using a 75 bp, single-end sequencing kit.

High-quality sequencing data were generated by removing low-quality and short (length <35 bp) reads, followed by computational subtraction of human host sequences mapped to the human reference genomes using Burrows-Wheeler alignment. The data remaining after removal of low-complexity reads were classified by simultaneous alignment to microbial genome databases consisting of viruses, bacteria, fungi, and parasites. The classification reference databases were downloaded from National Center Biotechnology Information (ftp://ftp.ncbi.nlm.nih.gov/genomes/).

2 Quality control of procedure

The process of sampling, storage, transportation and sequencing, aseptic procedures were strictly followed to ensure that qualified BALF was collected. In the lab, the specimens were kept in the -80 degree refrigerator to prevent nucleic acid degradation. All detection procedures are finished within 48 hours after receiving specimen ensuring timely guidance for clinical treatment.

Both Nucleic acid extraction and library preparation were conducted in parallel with quality control samples. And we compared the results of this whole process with the results analysed by database. The results of the two pipelines are highly consistent. To have the strict quality control system and automatically eliminating false-positive results, we used machine learning to perform simultaneous error modeling, denoising and exact sequence inference.

For these RNA mNGS of BALF, 97.48% reads were filtered for human genome respectively. And 420842 reads were mapped to the microbial genomes database respectively. The accuracy is 99.85%. The quality scores across all bases in the mNGS of BALF were showed as the figures below.

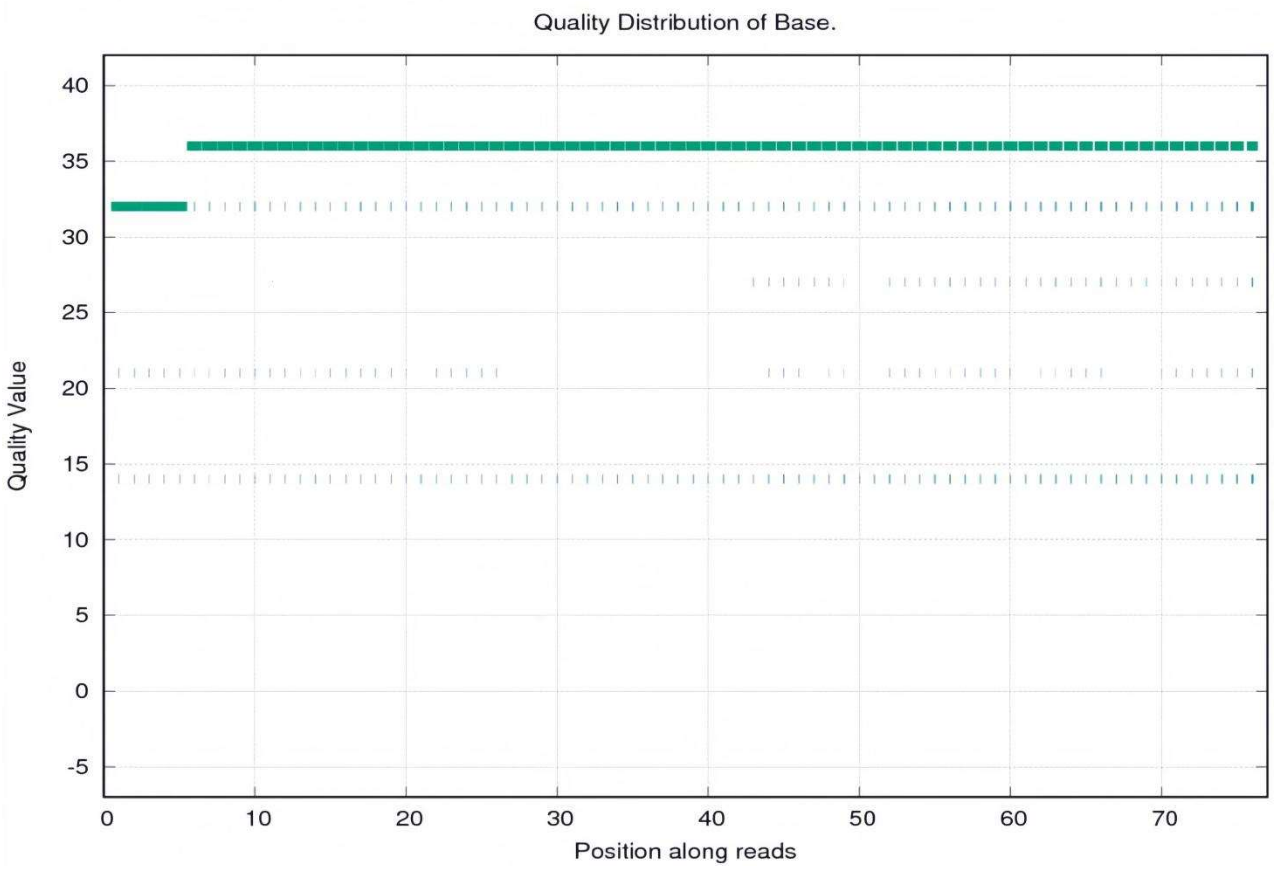

Supplementary Figure 1. The quality scores across all bases in the RNA mNGS of BALF.
